# Factors Associated with Untreated Diabetes: Analysis of Data from 20,496 Participants in the Japanese National Health and Nutrition Survey

**DOI:** 10.1371/journal.pone.0118749

**Published:** 2015-03-10

**Authors:** Maki Goto, Atsushi Goto, Nayu Ikeda, Hiroyuki Noda, Kenji Shibuya, Mitsuhiko Noda

**Affiliations:** 1 Department of Diabetes Research, Diabetes Research Center, National Center for Global Health and Medicine, Tokyo, Japan; 2 Center for International Collaboration and Partnership, National Institute of Health and Nutrition, Tokyo, Japan; 3 Public Health, Department of Social and Environmental Medicine, Graduate School of Medicine, Osaka University, Osaka, Japan; 4 Department of Global Health Policy, Graduate School of Medicine, University of Tokyo, Tokyo, Japan; University of Milan, ITALY

## Abstract

**Objective:**

We aimed to examine factors associated with untreated diabetes in a nationally representative sample of the Japanese population.

**Research Design and Methods:**

We pooled data from the Japanese National Health and Nutrition Survey from 2005 to 2009 (n = 20,496). Individuals aged 20 years and older were included in the analysis. We classified participants as having diabetes if they had HbA1c levels ≥6.5% (≥48 mmol/mol). People with diabetes who self-reported that they were not currently receiving diabetic treatment were considered to be untreated. We conducted a multinomial logistic regression analysis to determine factors associated with untreated diabetes relative to non-diabetic individuals.

**Results:**

Of 20,496 participants who were included in the analysis, untreated diabetes was present in 748 (3.6%). Among participants with untreated diabetes, 48.3% were previously diagnosed with diabetes, and 46.5% had HbA1c levels ≥7.0% (≥53 mmol/mol). Participants with untreated diabetes were significantly more likely than non-diabetic participants to be male, older, and currently smoking, have lower HDL cholesterol levels and higher BMI, non-HDL cholesterol levels, and systolic blood pressure.

**Conclusions:**

A substantial proportion of people in Japan with untreated diabetes have poor glycemic control. Targeting relevant factors for untreated diabetes in screening programs may be effective to enhance the treatment and control of diabetes.

## Introduction

Effective treatment coverage of diabetes is important to prevent its complications that increase the social cost of the disease. Diabetic complications reduce patients’ quality of life and increase the economic burden of diabetes [1,2]. The total cost associated with diabetes in the U.S. has increased from $174 billion in 2007 to $245 billion in 2012 [3].

In Japan, the estimated number of adults with suspected diabetes was approximately 9.5 million in 2012 [4] and diabetes accounts for 6% of the healthcare budget [5]. To promote the nation’s health, the central government initiated a 10-year campaign named “Health Japan 21 (the second term)” in 2013. In the campaign, four target goals were set for diabetes: 1) increasing the number of patients with diabetes receiving medical treatment; 2) reducing the number of patients with poor glycemic control; 3) reducing the number of new diabetic nephropathy hemodialysis cases; and 4) decreasing the incidence of newly diagnosed diabetes [4]. Improving the coverage of treatment for diabetes is a key, because as many as 35% of people who are strongly suspected of having diabetes are not receiving treatment in Japan [4].

In order to improve management of diabetes, it is essential to promote detection of diabetic patients who are not on treatment in the community. Information on characteristics of individuals who have untreated diabetes would help healthcare professionals in general practice and routine physical examinations distinguish them from those who are not diabetic. However, previous studies focused on undiagnosed diabetes, investigating the development and evaluation of diabetes screening tools, identification of significant factors for appropriate glycemic control, and documentation of diabetes-related complications [6–9]. We therefore aimed to identify characteristics of individuals with untreated diabetes compared to non-diabetic population in Japan.

## Materials and Methods

The National Health and Nutrition Survey (NHNS) has been conducted every November by the Ministry of Health, Labour and Welfare on a nationally representative sample of the population in Japan under the Health Promotion Law [10]. The survey started in 1947 as the National Nutrition Survey, and it was redesigned in 2003 to continue as the NHNS. After receiving permission for secondary use of survey data from the Ministry of Health, Labour and Welfare, we obtained access to anonymized individual-level data from participants who were surveyed between 2005 and 2009. This study was approved by the institutional review board (IRB) of the National Center for Global Health and Medicine. The requirement for informed consent was waived for this analysis by the IRB, because data were anonymized by the Ministry of Health, Labour and Welfare.

The survey aims at establishing measures for national health promotion and includes a cross-sectional interview and examination that obtain basic data on anthropometry, nutritional intake and diet, and lifestyle. Eligible respondents were all residents aged ≥1 year in a stratified random sample of 300 census tracts. Response rates of the NHNS are 60–70%, and the sample is considered representative of the Japanese population. A blood sample was taken from all participants aged 20 years and older [11–16].

HbA1c levels were measured using latex agglutination-turbidimetric immunoassay by SRL Inc., a commercial laboratory in Tokyo, Japan, which analyzed all of the NHNS blood samples [11]. HbA1c values were initially determined using Japan Diabetes Society (JDS) values, and we converted them to the National Glycohemoglobin Standardization Program (NGSP) values using the following conversion formula: HbA1c (NGSP) = 1.02 × HbA1c (JDS) + 0.25% [17]. We classified participants as having diabetes if they self-reported that they were currently receiving diabetes treatment or had HbA1c levels ≥6.5% (≥48 mmol/mol) [18]. We made no distinction between type 1 diabetes and type 2 diabetes. We defined untreated diabetes as participants who had diabetes and self-reported that they were not currently receiving diabetes treatment.

The subjects of the present study were adults aged ≥20 years. Participants were excluded from the analysis if they were pregnant, had missing HbA1c measurement values, or missing information for covariates or exposure variables.

In order to determine and analyze the characteristics of individuals with diabetes who were not receiving treatment (untreated diabetes), we compared this group and the group of respondents having diabetes who were on treatment (treated diabetes) with those who did not have diabetes (no diabetes). We used *t*-tests and Chi-squared tests to compare continuous and categorical baseline characteristics, respectively. We further conducted a multinomial logistic regression on the status of diabetes (untreated diabetes, treated diabetes, or non-diabetic) both for sexes combined and separately by sex. We treated the non-diabetic group as the base outcome of the dependent variable in the model. Independent variables of the regression model included sex (men/women), age (years), BMI (kg/m^2^), exercise habits (have exercise habits or have no exercise habit), smoking status (never, past, or current smoker), HDL cholesterol (mg/dL), non-HDL cholesterol (mg/dL), and systolic blood pressure (mmHg). We assessed factors for untreated diabetes against treated diabetes by using the estimated covariance matrix of regression coefficients to test the hypothesis that there was no difference in coefficients between untreated and treated diabetes.

All statistical tests were 2-sided and a *P*-value <0.05 was regarded as statistically significant. All statistical analyses were conducted using SAS (version 9.3; SAS Institute, Inc., Cary, NC, USA) and Stata software (version 12; Stata Corp, College Station, TX, USA).

## Results

**Fig 1:**
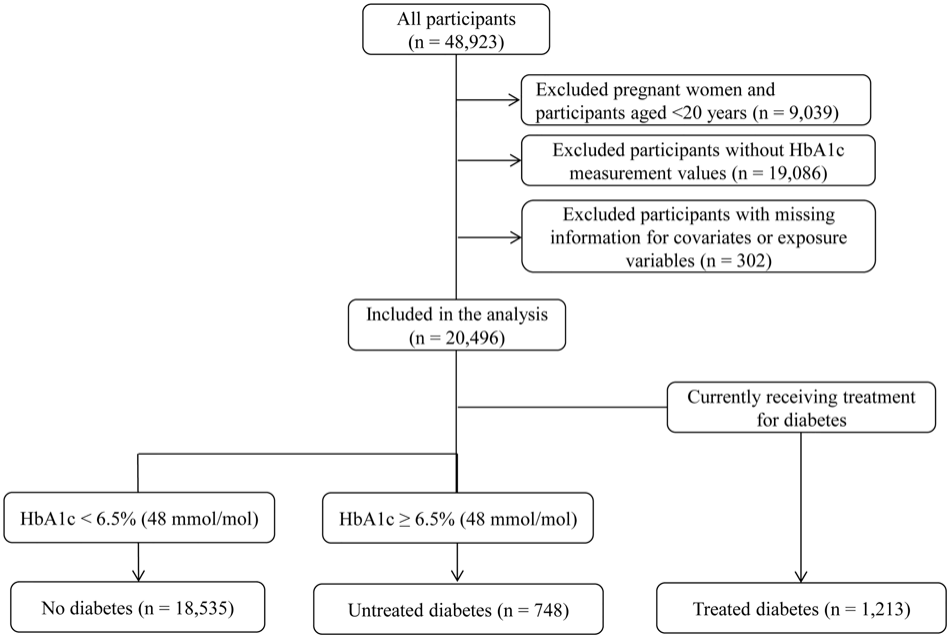
Flowchart of participant selection from the Japanese National Health and Nutrition Survey (NHNS). Abbreviations: HbA1c, hemoglobin A1c.

**Fig 2:**
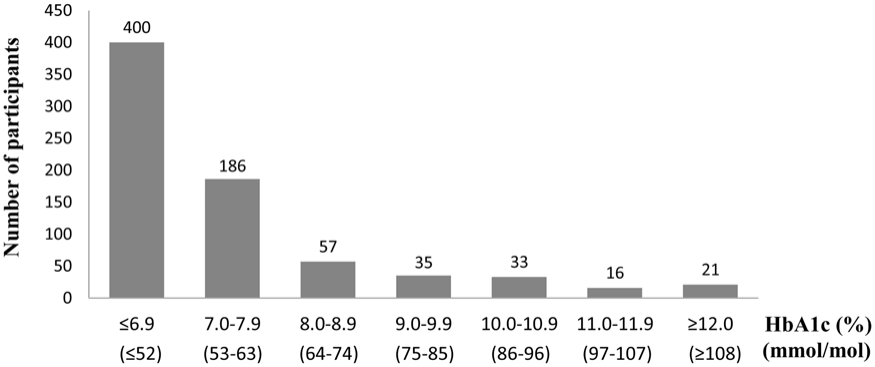
Distribution of HbA1c values among people with untreated diabetes (n = 748). Abbreviations: HbA1c, hemoglobin A1c.

Out of 20,496 participants included in this study, 748 (3.6%) had untreated diabetes and 1,213 (5.9%) had treated diabetes (Fig. 1). Compared to people without diabetes, people with untreated diabetes had a significantly larger proportion of men, had significantly higher age, HbA1c levels, BMI, total and non-HDL cholesterol levels, triglyceride levels, and blood pressure, had significantly lower HDL cholesterol levels, and reported significantly higher rates of exercise habits and past and current smoking (Table 1). Among the 748 people with untreated diabetes, 361 (48.3%) were previously diagnosed with diabetes, and 348 (46.5%) had HbA1c levels ≥7.0% [≥53 mmol/mol] (Fig. 2).

**Table 1:** Summary statistics of characteristics of participants included in the study by the status of diabetes.

Characteristics	No diabetes	Untreated diabetes	Treated diabetes	*P* value** (untreated vs. no diabetes)	*P* value**(treated vs. no diabetes)
	n = 18,535	n = 748	n = 1,213		
Men*	39.0	55.5	56.4	<0.001	<0.001
Age (years)	56.2 ± 16.6	64.9 ± 11.5	67.6 ± 9.8	<0.001	<0.001
HbA1c (%)	5.5 ± 0.4	7.6 ± 1.5	7.3 ± 1.3	<0.001	<0.001
	(37 ± 4 mmol/mol)	(60 ± 16 mmol/mol)	(56 ± 14 mmol/mol)		
BMI (kg/m^2^)	22.9 ± 3.4	25.1 ± 4.3	24.6 ± 3.7	<0.001	<0.001
Total cholesterol (mg/dL)	203.1 ± 34.8	211.3 ±38.2	198.9 ± 34.5	<0.001	<0.001
Triglycerides (mg/dL)	130.1 ± 88.4	188.1 ± 127.2	156.3 ± 102.5	<0.001	<0.001
HDL cholesterol (mg/dL)	62.7 ± 16.2	55.6 ± 15.1	56.5 ± 16.7	<0.001	<0.001
non-HDL cholesterol (mg/dL)	140.4 ± 35.5	155.8 ± 39.1	142.3 ± 35.0	<0.001	<0.001
Systolic blood pressure (mmHg)	131.0 ± 20.0	143.8 ± 19.9	141.4 ± 17.8	<0.001	<0.001
Diastolic blood pressure (mmHg)	79.3 ± 11.6	83.2 ± 12.3	79.1 ± 10.8	<0.001	<0.001
Have exercise habits*	28.8	33.3	42.9	0.01	<0.001
Past smoker*	19.5	25.3	26.4	<0.001	<0.001
Current smoker*	20.1	23.3	19.4	0.03	0.55

Results are presented as mean ± SD otherwise indicated. Abbreviations: HbA1c, hemoglobin A1c; BMI, body mass index; SD, standard deviation.

*Results are presented as percentages.

**P-values for the differences were computed by *t*-tests for continuous variables and by Chi-squared tests for categorical variables.

In the multinomial logistic regression for sexes combined, relative to the non-diabetic group, respondents with untreated diabetes were significantly more likely to be male (*P*-value < 0.001), older (*P*-value < 0.001), current smokers (*P*-value = 0.006), have a higher BMI (*P*-value < 0.001), non-HDL cholesterol level (*P*-value < 0.001), and systolic blood pressure (*P*-value < 0.001), and a lower HDL cholesterol (*P*-value = 0.005) (Table 2). For treated diabetes, similar associations were observed, although the association with current smoking was not significant (*P*-value = 0.063) and that with having exercise habits was significant (*P*-value < 0.001) (Table 2). Although the differences in the coefficients between untreated diabetes and treated diabetes were statistically significant for age (*P*-value < 0.001), exercise habits (*P*-value < 0.001), non-HDL cholesterol (*P*-value < 0.001), and systolic blood pressure (*P*-value = 0.002), most factors exhibited directional consistency in the sign of the coefficients, with the exception of non-HDL cholesterol. Although the results were similar in the analyses stratified by sex, the positive association of current smoking with untreated diabetes was significant only in men, and the inverse associations of HDL cholesterol with treated and untreated diabetes were significant only in women (Table 2).

**Table 2:** Factors associated with untreated and treated diabetes vs. no diabetes.

	Total		Men		Women	
	n = 20,496		n = 8,326		n = 12,170	
	Untreated diabetes	Treated diabetes	Untreated diabetes	Treated diabetes	Untreated diabetes	Treated diabetes
Independent variables						
Sex (men)	1.46 (1.21, 1.78)	1.53 (1.32, 1.79)				
Age (in 10-year increments)	1.38 (1.30, 1.47)	1.60 (1.52, 1.69)	1.57 (1.44, 1.72)	1.60 (1.49, 1.71)	1.15 (1.05, 1.26)	1.57 (1.45, 1.70)
BMI (kg/m^2^)	1.13 (1.11, 1.16)	1.13 (1.11, 1.15)	1.11 (1.07, 1.14)	1.10 (1.08, 1.13)	1.15 (1.12, 1.18)	1.14 (1.12, 1.70)
Have exercise habits	1.10 (0.94, 1.29)	1.58 (1.40, 1.78)	1.08 (0.87, 1.33)	1.62 (1.37, 1.91)	1.12 (0.88, 1.18)	1.55 (1.29, 1.87)
Past smoker	1.19 (0.96, 1.47)	1.07 (0.90, 1.27)	1.15 (0.89, 1.49)	1.07 (0.88, 1.30)	1.23 (0.81, 1.43)	1.04 (0.71, 1.53)
Current smoker	1.37 (1.10, 1.71)	1.19 (0.99, 1.43)	1.57 (1.20, 2.06)	1.19 (0.96, 1.48)	0.90 (0.57, 1.89)	1.18 (0.81, 1.71)
HDL cholesterol (in 10 mg/dL increments)	0.92 (0.87, 0.98)	0.91 (0.87, 0.95)	0.99 (0.92, 1.07)	0.96 (0.91, 1.02)	0.84 (0.77, 0.91)	0.84 (0.79, 0.90)
Non-HDL cholesterol (in 10 mg/dL increments)	1.07 (1.05, 1.09)	0.97 (0.95, 0.99)	1.07 (1.04, 1.10)	0.97 (0.95, 0.997)	1.07 (1.04, 1.11)	0.96 (0.94, 0.99)
Systolic blood pressure (in 10 mmHg increments)	1.17 (1.12, 1.21)	1.08 (1.04, 1.12)	1.12 (1.06, 1.18)	1.04 (0.99, 1.08)	1.23 (1.16, 1.30)	1.13 (1.07, 1.19)

Results are presented as adjusted RPR (95% CI). A multinomial logistic regression analysis was used to estimate adjusted RPRs with the independent variables in the table. Abbreviations: BMI, body mass index; CI, confidence interval; HDL, high-density lipoprotein; RPR, ratio of prevalence ratio.

## Discussion

This study explored factors that are associated with untreated diabetes in the Japanese population. The likelihood of having untreated diabetes increased in the presence of male sex, older age, higher BMI, current smoking status, decreased HDL cholesterol levels, and increased non-HDL cholesterol and systolic blood pressure levels. Using a multinomial logistic regression analysis, we compared the findings for untreated and treated diabetes and observed directional consistency for all factors, with the exception that non-HDL cholesterol was positively associated with untreated diabetes and inversely associated with treated diabetes. The frequent use of anti-hyperlipidemic agents among participants who were being treated for diabetes may have improved their serum lipid control, which may explain the difference in direction of the association.

Previous studies have focused on the factors associated with undiagnosed diabetes [7,19,20], but not the factors for untreated diabetes. Although the factors that are associated with untreated and undiagnosed diabetes may differ, it is relevant to compare our results with previous studies that examined the factors associated with undiagnosed diabetes. In a systematic review and meta-analysis of studies that examined screening scores to detect undiagnosed diabetes, age and adiposity measures (e.g., BMI) were the most commonly used factors to detect undiagnosed diabetes [19]. This result is consistent with our observation that age and BMI were strongly and positively associated with untreated diabetes. Other factors that were identified by our study, such as sex, current smoking, hypertension, and exercise habits, were also commonly used to detect undiagnosed diabetes. In previous studies, family history of diabetes was another strong predictor of undiagnosed diabetes [7,19], although this information was not available for our study. Although decreased HDL cholesterol and increased non-HDL cholesterol levels were predictors of untreated diabetes in our study, lipid levels have rarely been used to detect undiagnosed diabetes [7,19], possibly because blood testing is needed to evaluate lipid levels.

In our study, the association between untreated diabetes and current smoking status was observed only in men. This may be explained by the fact that the proportion of current smokers in the group of untreated diabetes was lower in women (6.6% [22/333]) than in men (36.6% [152/415]). Our study also showed that the inverse association between untreated diabetes and HDL cholesterol was observed only in women. This result is supported by findings in the follow-up of the Finnmark study, which reported that HDL cholesterol was a strong independent risk factor of diabetes in women, but not in men [21]. Although possible mechanisms responsible for the sex-difference remain to be examined, the difference may reflect effects of sex hormones on glucose and lipid metabolism [22,23].

Our findings support the notion that selective or targeted screening programs performed in a subgroup with factors such as male sex, older age, and higher BMI may be effective to reduce the proportion of untreated diabetes. Further, our results indicated that as many as half of people with untreated diabetes had previously been diagnosed with diabetes; that is, treatment did not follow diagnosis in these participants. However, it remains uncertain which environments are most suitable to provide motivation for people to access medical services.

The major strength of this study is the use of nationwide data that represents the Japanese population. However, some limitations of this study need to be addressed. First, although the response rate was relatively high [14], the risk for selection and reporting bias may still exist. Second, additional information about a person’s history of diabetes, such as family history, duration, and complications, was not available from the NHNS. Third, we excluded participants who had a missing value on HbA1c, potentially resulting in selection bias. Finally, we were unable to establish from this cross-sectional analysis a temporal relationship required for causality, and the results need to be interpreted cautiously.

In conclusion, in Japan, untreated diabetes were associated with male sex, current smoking, older age, higher BMI, higher non-HDL cholesterol levels, higher systolic blood pressure, and lower HDL cholesterol levels. A substantial proportion of people with untreated diabetes are previously diagnosed with diabetes and have poor glycemic control. Our findings support the notion that selective or targeted screening programs performed in a subgroup with factors that were associated with a lack of treatment in diabetes may be effective to reduce the proportion of untreated diabetes.

## References

[1] Huang ES, Brown SE, Ewigman BG, Foley EC, Meltzer DO (2007) Patient perceptions of quality of life with diabetes-related complications and treatments. Diabetes Care 30: 2478–2483. 17623824 10.2337/dc07-0499. PMC2288662

[2] Clarke P, Gray A, Legood R, Briggs A, Holman R (2003) The impact of diabetes-related complications on healthcare costs: results from the United Kingdom Prospective Diabetes Study (UKPDS Study No. 65). Diabetic Medicine 20: 442–450. 12786677 10.1046/j.1464-5491.2003.00972.x

[3] Herman WH (2013) The economic costs of diabetes: is it time for a new treatment paradigm? Diabetes Care 36: 775–776. 10.2337/dc13-0270 23520368 PMC3609514

[4] Ministry of Health, Labour and Welfare (2012) Reference data of Healthy Japan 21 (Second campaign) (in Japanese). Available: http://www.mhlw.go.jp/bunya/kenkou/dl/kenkounippon21_02.pdf.

[5] Neville SE, Boye KS, Montgomery WS, Iwamoto K, Okamura M, Hayes RP. (2009) Diabetes in Japan: a review of disease burden and approaches to treatment. Diabetes Metab Res Rev 25: 705–716. 10.1002/dmrr.1012 19795421

[6] Tekumit H, Cenal AR, Polat A, Uzun K, Tataroglu C, Akinci E. (2010) Diagnostic value of hemoglobin A1c and fasting plasma glucose levels in coronary artery bypass grafting patients with undiagnosed diabetes mellitus. Ann Thorac Surg 89: 1482–1487. 10.1016/j.athoracsur.2009.11.033 20417764

[7] Heianza Y, Arase Y, Saito K, Hsieh SD, Tsuji H, Kodama S, et al (2013) Development of a screening score for undiagnosed diabetes and its application in estimating absolute risk of future type 2 diabetes in Japan: Toranomon Hospital Health Management Center Study 10 (TOPICS 10). J Clin Endocrinol Metab 98: 1051–1060. 10.1210/jc.2012-3092 23393174

[8] Lauruschkat AH, Arnrich B, Albert AA, Walter JA, Amann B, Rosendahl UP, et al (2005) Prevalence and risks of undiagnosed diabetes mellitus in patients undergoing coronary artery bypass grafting. Circulation 112: 2397–2402. 16230496 10.1161/CIRCULATIONAHA.105.534545

[9] Choi KM, Lee KW, Kim SG, Kim NH, Park CG, Seo HS, et al (2005) Inflammation, insulin resistance, and glucose intolerance in acute myocardial infarction patients without a previous diagnosis of diabetes mellitus. J Clin Endocrinol Metab 90: 175–180. 15509644 10.1210/jc.2004-1795

[10] Ministry of Health, Labour and Welfare (2011) The National Health and Nutrition Survey, 2009. Tokyo: Ministry of Health, Labour and Welfare.

[11] Nakamura M, Kiyama M, Kitamura A, Ishikawa Y, Sato S, Noda H, et al (2013) Revised system to evaluate measurement of blood chemistry data from the Japanese National Health and Nutrition Survey and Prefectural Health and Nutrition Surveys. J Epidemiol 23: 28–34. 23117223 10.2188/jea.JE20120032 PMC3700228

[12] Tokudome S, Nishi N, Tanaka H (2012) Towards a better National Health and Nutrition Survey in Japan. Lancet 379: e44 10.1016/S0140-6736(12)60466-8 22444403

[13] Katanoda K, Nitta H, Hayashi K, Matsumura Y (2005) Is the national nutrition survey in Japan representative of the entire Japanese population? Nutrition 21: 964–966. 16039832 10.1016/j.nut.2005.02.004

[14] Yoshiike N, Matsumura Y, Yamaguchi M, Seino F, Kawano M, Inoue K, et al (1998) Trends of average intake of macronutrients in 47 prefectures of Japan from 1975 to 1994—possible factors that may bias the trend data. J Epidemiol 8: 160–167. 9782672 10.2188/jea.8.160

[15] Ikeda N, Gakidou E, Hasegawa T, Murray CJ (2008) Understanding the decline of mean systolic blood pressure in Japan: an analysis of pooled data from the National Nutrition Survey, 1986–2002. Bulletin of the World Health Organization 86: 978–988. 19142299 10.2471/BLT.07.050195 PMC2649578

[16] Katanoda K, Matsumura Y (2002) National Nutrition Survey in Japan ―its methodological transition and current findings―. J Nutr Sci Vitaminol (Tokyo) 48: 423–432. 12656220 10.3177/jnsv.48.423

[17] Kashiwagi A, Kasuga M, Araki E, Oka Y, Hanafusa T, Ito H, et al (2012) International clinical harmonization of glycated hemoglobin in Japan: From Japan Diabetes Society to National Glycohemoglobin Standardization Program values. J Diabetes Investig 3: 39–40. 10.1111/j.2040-1124.2012.00207.x 24843544 PMC4014931

[18] International Expert Committee (2009) International Expert Committee report on the role of the A1C assay in the diagnosis of diabetes. Diabetes Care 32: 1327–1334. 10.2337/dc09-9033 19502545 PMC2699715

[19] Brown N, Critchley J, Bogowicz P, Mayige M, Unwin N (2012) Risk scores based on self-reported or available clinical data to detect undiagnosed type 2 diabetes: a systematic review. Diabetes Res Clin Pract 98: 369–385. 10.1016/j.diabres.2012.09.005 23010559

[20] Lee YH, Bang H, Kim HC, Kim HM, Park SW, Kim DJ. (2012) A simple screening score for diabetes for the Korean population: development, validation, and comparison with other scores. Diabetes Care 35: 1723–1730. 10.2337/dc11-2347 22688547 PMC3402268

[21] Njolstad I, Arnesen E, Lund-Larsen PG (1998) Sex differences in risk factors for clinical diabetes mellitus in a general population: a 12-year follow-up of the Finnmark Study. Am J Epidemiol 147: 49–58. 9440398 10.1093/oxfordjournals.aje.a009366

[22] Vaidya D, Dobs A, Gapstur SM, Golden SH, Hankinson A, Liu K, et al (2008) The association of endogenous sex hormones with lipoprotein subfraction profile in the Multi-Ethnic Study of Atherosclerosis. Metabolism 57: 782–790. 10.1016/j.metabol.2008.01.019 18502260 PMC4017356

[23] Ding EL, Song Y, Malik VS, Liu S (2006) Sex differences of endogenous sex hormones and risk of type 2 diabetes: a systematic review and meta-analysis. JAMA 295: 1288–1299. 16537739 10.1001/jama.295.11.1288

